# Inhibitory Potential of the Drimane Sesquiterpenoids Isotadeonal and Polygodial in the NF-kB Pathway

**DOI:** 10.3390/molecules30071555

**Published:** 2025-03-31

**Authors:** Víctor Marín, Cecilia Villegas, Ayorinde Víctor Ogundele, Jaime R. Cabrera-Pardo, Bernd Schmidt, Cristian Paz, Viviana Burgos

**Affiliations:** 1 Laboratory of Natural Products & Drug Discovery, Center CEBIM, Department of Basic Sciences, Faculty of Medicine, Universidad de La Frontera, Temuco 4780000, Chile; victor.marinmosi.bq@gmail.com (V.M.); vicshow2001@gmail.com (A.V.O.); 2 Departamento de Ciencias Biológicas y Químicas, Facultad de Recursos Naturales, Universidad Católica de Temuco, Rudecindo Ortega, Temuco 4780000, Chile; cecivi42@hotmail.com; 3 Laboratorio de Química Aplicada y Sustentable (LabQAS), Departamento de Química, Universidad del Bío-Bío, Avenida Collao 1202, Concepcion 4051381, Chile; jacabrera777@gmail.com; 4 College of Dental Medicine, Roseman University of Health Sciences, 10894 S. River Front Parkway, South Jordan, UT 84095, USA; 5 Institut für Chemie, Universität Potsdam, Karl-Liebknecht-Str. 24-25, D-14476 Potsdam, Germany; bernd.schmidt@uni-potsdam.de; 6 Escuela de Tecnología Médica, Facultad de Salud, Universidad Santo Tomás, Temuco 4780000, Chile

**Keywords:** drimane sesquiterpenoids, polygodial, isotadeonal, *Drimys winteri*, NF-κB

## Abstract

Inflammation contributes to the onset and development of many diseases, including neurodegenerative diseases, caused by the activation of microglia, leading to neurological deterioration. Nuclear factor-κB (NF-κB) is one of the most relevant pathways for identifying anti-inflammatory molecules. In this study, polygodial and isotadeonal, two drimane sesquiterpene dialdehydes, were isolated from *Drimys winteri*, a medicinal tree of the Mapuche people in Chile. Isotadeonal, or epi-polygodial, was obtained from polygodial by epimerization in basic media (60% yield, Na_2_CO_3_, r/t, 24 h). Both sesquiterpenoids were evaluated on the NF-κB pathway, with the result that isotadeonal inhibited the phosphorylation of IκB-α at 10 μM with higher potency by Western blotting. The final inhibition of the pathway was evaluated using a SEAP reporter (secreted alkaline phosphatase) on THP-1 cells. Isotadeonal inhibited SEAP with higher potency than polygodial, quercetin, and CAPE (phenethyl ester of caffeic acid). In silico analysis suggests that the α-aldehyde of isotadeonal adopts a more stable conformation in the active pocket of IκB-α than polygodial.

## 1. Introduction

NF-κB consists of a group of transcription factors that regulate immune and inflammatory responses. Several signal transduction cascades can activate the NF-κB pathway, resulting in the translocation of NF-κB proteins from the cytoplasm to the nucleus, where they activate the expression of specific cellular genes [1]. NF-κB activation involves two main signaling pathways: the canonical and the non-canonical pathways. Canonical NF-κB activation involves the degradation of IκBα through phosphorylation by a multi-subunit IκB kinase (IKK) [2]. IKK is composed of two catalytic subunits, IKKα and IKKβ, and a regulatory subunit, IKKγ. IKK can be activated by different stimuli, including cytokines, growth factors, mitogens, microbial components, and stress agents [3,4]. Many diseases begin with an inflammatory development, including central nervous system diseases such as Alzheimer’s and Parkinson’s. Neuroinflammation is described as the stimulation of the immune system in the central nervous system, which results in a greater release of chemical mediators [5]. Natural products with inhibitory activity against NF-κB could be valuable in the treatment of inflammatory diseases. Among these, quercetin and the phenethyl ester of caffeic acid (CAPE) are two commercially available NF-κB inhibitors [6,7]. In spite of the broad activity of these compounds in cells and in animal models, the bioavailability of these polyphenols in the central nervous system is still controversial due to their fast excretion and poor permeability through the blood–brain barrier (BBB) [8,9].

Drimys winteri or Canelo is a medicinal plant native to Chile that has been traditionally used by the Mapuche people, who consider it a sacred tree, a symbol of benevolence, peace, and justice. Nowadays, it is present in all social and religious meetings called “guillatún” and “machitún”, where the healer, called “machi”, uses Canelo leaves or sap as medicine [10]. Known properties of Canelo or its secondary metabolites include insecticidal, bactericidal activity with biofilm inhibition by quorum sensing [11,12], trypanocidal activity [13], and fungicidal effects against fungi such as Gaeumannomyces graminis, which affect crops [14], or for the control of Candida species by the inhibition of the enzyme lanosterol 14-alpha demethylase [15].

The medicinal activity of Drimys winteri is due to a set of chemical compounds found in bark and leaves, identified as drimane sesquiterpenes. Polygodial is a 1,4-dialdehyde sesquiterpene, a pungent compound that acts by activating two members of the Transient receptor potential ankyrin 1 (TRPA1) and the Transient receptor vanilloid 1 (TRPV1), which are both nonselective calcium-permeable cation channels expressed in nociceptive neurons. These two channels also play a role in pain perception [16,17]. The anti-inflammatory effects of polygodial are varied, including the inhibition of Intercellular adhesion molecule 1 (ICAM1) and Vascular cell adhesion molecule 1 (VCAM1), which are involved in endothelial dysfunction [18]. The anti-inflammatory effect of polygodial was similar to the glucocorticoid dexamethasone, increasing the expression of MKP-1 and decreasing the expression of ERK1/2 in a dose-response and time-dependent manner, interacting at the ligand-binding domain of the glucocorticoid receptor [19].

**Scheme 1:**
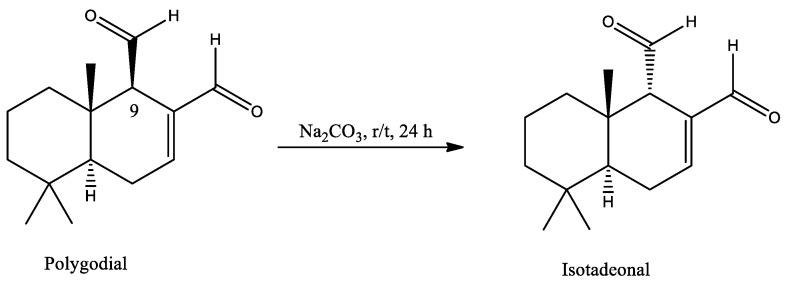
Epimerization of polygodial to isotadeonal at the stereogenic center C9.

Isotadeonal, also called epi-polygodial, is a natural analogue of polygodial and is also present in the organic extract of Drimys winteri. Polygodial and isotadeonal differ only in the configuration at C9 (Scheme 1). Less information is available about isotadeonal than for polygodial; a Google Scholar search gives 101 results for the entry isotadeonal, compared to 3950 results for the entry polygodial. The effects reported for this compound are different compared to polygodial. It has antifungal and fungistatic activity against Candida yeast comparable to polygodial, but without irritating effects on eyes and skin [20], and exhibits mild antibacterial activity (MIC 2–10 µg/mL) [21]. Herein, the inhibitory activity of isotadeonal and polygodial on the NF-κB pathway is reported, comparing the molecular structure of both drimane sesquiterpenoids from in vitro and in silico studies. The final pathway was evaluated by a reporter cell (THP-1/SEAP) that produces alkaline phosphatase in response to NF-κB activation, finding that isotadeonal inhibits the pathway with higher potency than polygodial and positive controls like CAPE and quercetin. Moreover, isotadeonal inhibits the phosphorylation of IkB-*α* in microglia cells (HMC-3), which could explain this activity.

## 2. Results

### 2.1. Epimerization of Polygodial

Polygodial was isolated from a concentrated fraction obtained by extracting Drimys winteri bark with ethyl acetate (EtOAc). The concentrate was purified by column chromatography (CC) on silica with hexane/EtOAc (4:1 *v*/*v*) as the eluent, monitored by thin layer chromatography (TLC) and visualized by UV (254 nm), and stained with KMnO_4_. A total of 345 mg of polygodial was purified, and then 100 mg was used to obtain isotadeonal by epimerization in basic media with sodium carbonate, producing 60 mg of isotadeonal after CC. Isotadeonal showed a retention factor (R_f_) of 0.68, compared to polygodial, which had an R_f_ of 0.54 in hexane/EtOAc (4:1 *v*/*v*) (Scheme 1).

The NMR data of polygodial and isotadeonal match those previously reported in the literature [22]. Copies of the spectra and a detailed signal assignment are available as Supplementary Material.

### 2.2. Cell Viability

**Figure 1:**
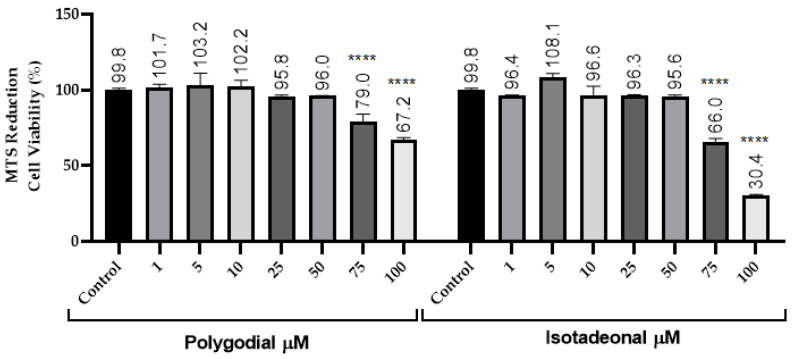
Effect of polygodial and isotadeonal on the cell viability of HMC-3. Cell viability was assessed by MTS assay compared to control (cells without treatment). The data are expressed as mean ± standard deviation. ANOVA, Dunnett’s Multiple Comparison Test (**** = *p* < 0.0001 vs. Control).

The cell viability of polygodial and isotadeonal was evaluated by MTS assays over 24 h, using concentrations between 1 and 100 μM in HMC-3. Regarding experimental treatments, polygodial and isotadeonal showed a significant decrease in cell viability at concentrations of 75 μM, decreasing the cell viability below 70% (*p* < 0.001, Figure 1). Considering these data, concentrations up to 50 μM were not toxic to the cells. In this work, both compounds were used at 10 and 50 μM, ensuring a viability close to 100%.

### 2.3. Drimane Sesquiterpenoids Modulate the Expression of NF-kB-SEAP

The inhibition of the NF-κB signaling pathway was evaluated in THP-1 reporter cells exposed to polygodial and isotadeonal at concentrations between 1 and 50 µM, using SEAP production as an activity marker. The NF-κB pathway was stimulated with LPS (100 ng/mL), and then quercetin and CAPE were used as positive controls, because they are potent anti-inflammatory compounds acting by inhibition of NF-κB [23]. Polygodial at 25 µM showed a significant decrease in SEAP (*p* < 0.0001) of 56.9%, while at 50 µM, it was reduced to 52.1% (*p* < 0.0001), indicating dose-dependent inhibition, comparable in potency to quercetin and higher than CAPE.

**Figure 2:**
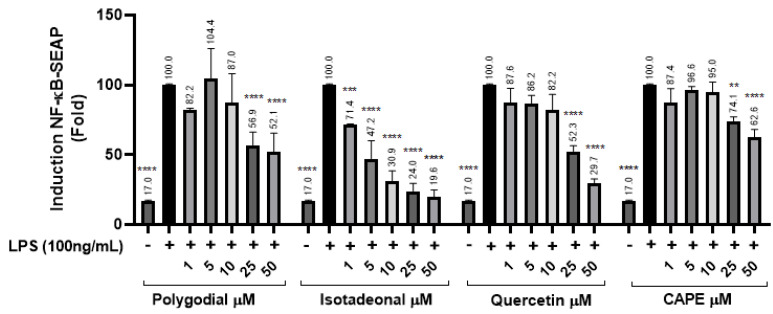
Effects of polygodial and isotadeonal on the induction of NF-κB-SEAP. THP1-Dual^TM^ cells were treated with each compound (1–50 μM) and LPS (100 ng/mL) for 18 h and assessed by the THP-1-Dual^TM^ cell assay, compared to positive control LPS (100 ng/mL). The data are expressed as mean ± standard deviation. ANOVA, Dunnett’s Multiple Comparison Test. (** *p* = < 0.01; *** *p* = 0.001, and **** = *p* < 0.0001 vs. LPS (+) group).

Moreover, isotadeonal showed better activity than polygodial, with a significant inhibition of SEAP to 71.4% (*p* < 0.001) at 1 µM. These results suggest that both compounds inhibit the NF-κB pathway in a concentration-dependent manner, with isotadeonal being more effective than polygodial, as well as both quercetin and CAPE, two known anti-inflammatory molecules (Figure 2).

### 2.4. Drimane Sesquiterpenoids Modulate the Expression of IkB-α

**Figure 3:**
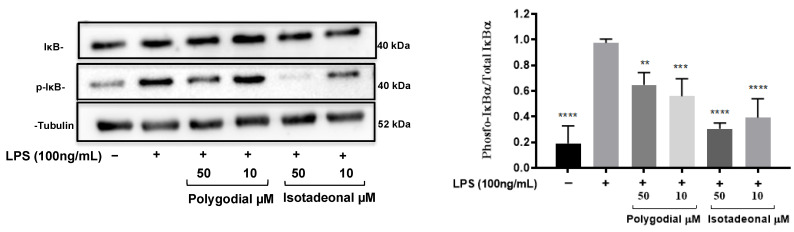
Effect of polygodial and isotadeonal on the protein expression of IkB-*α/*p-IkB-*α* in stimulated HMC-3 cells with LPS. HMC-3 cells were treated with polygodial and isotadeonal at 50 µM and 10 µM, as well as LPS (100 ng/mL), for 4 h. Protein expression of IkB-*α*, p-IkB-*α,* and *α*-tubulin was detected by Western blot analysis, normalized to the positive control group LPS (100 ng/mL). Data are expressed as mean ± standard deviation. ANOVA, Dunnett`s Multiple Comparison Test. (** = *p* < 0.01; *** *p* = < 0.001 and **** = *p* < 0.0001 vs. LPS (+) group).

The effect of polygodial and isotadeonal on the inhibition of NF-κB was evaluated by Western blot in HMC-3 cells stimulated with LPS (100 ng/mL) as a result of the inhibition of phosphorylated IkB-*α* (p-IkB-*α*), Figure 3. A similar pattern to the expression of NF-κB-SEAP was observed with the protein expression of IkB-*α*. Isotadeonal and polygodial (10 and 50 µM) significantly decreased the levels of p-IkB-*α* compared to the expression of IkB-*α*, after LPS stimulation, Figure 3. On the other hand, the protein levels decreased with greater significance (*p* < 0.001) with isotadeonal compared to polygodial, at the same concentrations. These results suggest that both compounds inhibit the NF-κB pathway by preventing IκBα phosphorylation and the release of the p50/p65 dimer.

### 2.5. Molecular Docking

NF-κB is a key transcription factor regulating inflammation, immunity, and cell survival, and is activated when IKKβ phosphorylates its inhibitor, IκB. This activation is linked to cancer and inflammatory diseases. Targeting IKKβ with natural inhibitors is a promising strategy to safely block NF-κB signaling and manage related conditions [24].

To validate our experimental findings, a molecular docking analysis was performed to investigate the binding affinity, interaction types, and interactions between the examined compounds and the binding pockets of the target protein. The outcomes of the docking study are presented in Table 1 and Table 2. The molecular docking of polygodial and isotadeonal was performed in the binding pocket of IKKβ in PDB: 4KIK. This was done to gain insight into the binding mode and rationalize the observed in vitro activity of the compounds. Table 1 provides details on binding energies and interaction parameters, such as the number of interactions, interacting amino acids, and hydrogen bonds formed between the ligands and the target protein. The tested compounds were successfully docked within the active site, displaying varying scores and binding interactions with the receptor pocket’s amino acids. Based on the docking results summarized in Table 1, isotadeonal showed the highest theoretical binding affinity against IKKβ of −7.12 kcal/mol, while polygodial displayed a score of −6.68 kcal/mol, targeting the same three residues within the active site of IKKβ. Interestingly, these findings align with the experimental results, which revealed greater activity for isotadeonal compared to polygodial.

The interaction diagrams of IKKβ (PDB: 4KIK) complexed with isotadeonal and polygodial are shown in Table 2. Cys99 has been identified as a pivotal residue for inhibitor binding within the ATP-binding site of IKKβ, playing a critical role in its inhibition [25]. Binding of bioactive agents to this residue is essential for effective inhibition of the protein. According to our results, polygodial has a distance of 2.75 Å, while isotadeonal showed a distance of 2.64 Å. Isotadeonal formed additional interactions with Val152 and Leu21. Polygodial displayed comparable interactions with the same residues; however, the additional alkyl bond interaction observed between isotadeonal and Cys99, compared to the single hydrogen bond interaction in polygodial, likely accounts for the slightly higher binding affinity and superior activity of isotadeonal.

### 2.6. Pharmacokinetic Features—ADMET

**Figure 4:**
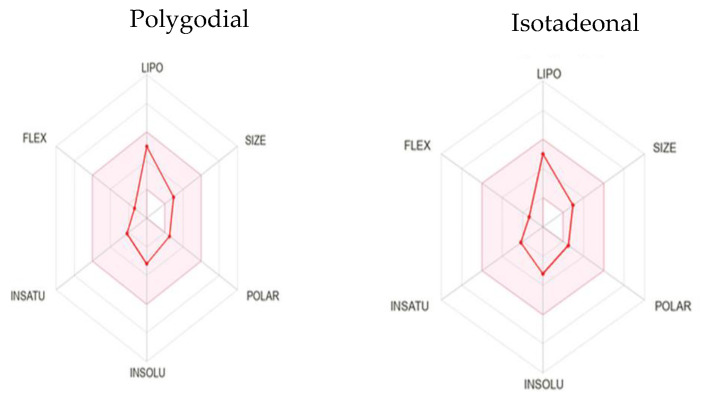
Bioavailability radar of polygodial and isotadeonal.

The in silico pharmacokinetic predictions of the compounds, presented in Table 3 and visualized through bioavailability radars in Figure 4, provide a comparative assessment of their physicochemical profiles.

These predictions offer valuable insights into their ADMET (Absorption, Distribution, Metabolism, Excretion, and Toxicity) properties and overall druggability. Both compounds comply with Lipinski’s Rule of Five without any violations, suggesting favorable oral bioavailability. This is indicated by their molecular weight (MW) < 500 Da, number of rotatable bonds (RB) < 2, hydrogen bond acceptors (HBA) < 2, hydrogen bond donors (HBD) = 0, and partition coefficient (log P) < 3, supporting their promising physicochemical and pharmacokinetic properties for drug development [26].

## 3. Discussion

Microglia-mediated neuroinflammation plays a crucial role in the onset and development of central nervous system diseases such as Alzheimer disease, where reactive oxygen species (ROS), amyloid peptides, hyperphosphorylated tau protein, and neurofibrillary tangles are overexpressed. With neuroinflammation playing a pivotal role in the development and onset of dementia, this topic has been reviewed by many authors [27,28,29]. Microglia cells are innate immune cells of the central nervous system that can be activated to the M1 phenotype, which is responsible for neuronal damage by releasing proinflammatory cytokines [30]. Inflammatory signaling pathways such as NF-κB are overexpressed in Alzheimer’s patients [31]. NF-κB can be activated by inflammatory mediators like LPS and its product TNFα, which promote the degradation of the IKK complex (IKKα, IKKβ, and the regulatory subunit IKKγ) by phosphorylation-ubiquitination and proteasomal degradation. This process releases NF-κB dimers (p50–p65), which can translocate to the nucleus, stimulating the production of proinflammatory cytokines like TNFα, IL1β, and IL6, among others [32].

In this work, we studied two drimane sesquiterpenes, polygodial and isotadeonal, obtained from the Chilean medicinal tree *Drimys winteri*. In contrast to polygodial, which was isolated from this tree in major amounts, isotadeonal was produced by epimerization of polygodial. The structures of both compounds, polygodial and isotadeonal, were elucidated by 1D and 2D NMR spectroscopy. They did not show toxic effects on cell viability up to a concentration of 50 μM (Figure 1).

The possible anti-inflammatory effects were screened by THP-1 SEAP reporter cells, where isotadeonal showed notable inhibition of SEAP production from 1 to 50 μM (71.4 and 19.6%, respectively), followed by polygodial, which decreased SEAP at 25 and 50 μM (56.9% and 52.1%, respectively), while the positive controls quercetin and CAPE [33] showed lower activity at the same concentrations. In addition to these results, polygodial and isotadeonal prevented canonical NF-κB activation in HMC-3 by regulating the phosphorylation of IκBα (Figure 3). This activity had greater significance for isotadeonal (*p* < 0.0001, Figure 3) than polygodial at 10 μM. In silico studies suggest that the observed differences can be attributed to subtle stereochemical and conformational variations between polygodial and isotadeonal, which likely influence their binding interactions with IKKβ. Isotadeonal seems to adopt a conformation that optimally engages the active site, resulting in its slightly higher binding affinity. This suggests the possibility of robust inhibitory binding to IKKβ, as experimentally confirmed by the data presented in Table 1. These compounds showed advantages in blood–brain barrier (BBB) permeability compared to polyphenols, such as quercetin and CAPE. Higher solubility is a key factor influencing drug absorption and distribution, while enhanced BBB permeability suggests their potential for neurological applications, making them promising candidates for targeting central nervous system (CNS) disorders [34]. The predicted topological polar surface area (TPSA) values for the compounds are 34.14 Å^2^, which is outside the preferred range of 70–140 Å^2^ [35]. However, this deviation may not significantly affect their druggability, as several established NF-κB pathway inhibitors, such as carfilzomib (TPSA = 158.47 Å^2^) and doxorubicin (TPSA = 206.07 Å^2^), have considerably higher TPSA values, but still remain effective. Notably, carfilzomib, a proteasome inhibitor, exerts its effect by irreversibly binding to the 20S proteasome, thereby disrupting NF-κB signaling through the inhibition of IκB degradation [36]. On the other hand, doxorubicin, a topoisomerase II inhibitor, primarily induces DNA intercalation and oxidative stress, leading to the suppression of NF-κB activation [37]. Despite their different mechanisms of action, both compounds effectively modulate NF-κB signaling, highlighting that TPSA alone does not strictly determine drug efficacy. While polygodial and isotadeonal exhibit promising pharmacological potential, their aldehyde group and Michael acceptor moieties are structural alerts in medicinal chemistry, as they may react with biological nucleophiles like cysteine, leading to off-target effects or toxicity [38]. The presence of Cys99 in the ATP-binding site of IKKβ raises the possibility of covalent interactions, which could enhance binding but also increase the risk of irreversible modification. Structural modifications, such as converting the aldehyde to a less reactive functional group or developing prodrugs, may improve selectivity while mitigating toxicity concerns. Experimental validation through covalent docking and biochemical assays (e.g., protein NMR) could further elucidate the nature of these interactions. Moreover, these compounds exhibited no inhibitory effects on key cytochrome P450 (CYP) enzymes, suggesting a favorable metabolic profile with lower toxicological risks. Further studies should focus on structural refinement, in vivo pharmacokinetics, and toxicity assessments to optimize their therapeutic viability.

## 4. Materials and Methods

### 4.1. Cell Culture

Microglial cells (HMC-3) were obtained from the American Type Culture Collection (ATCC) and maintained in DMEM/High Glucose (Cytiva, Marlborough, UK) supplemented with 4 mM l-glutamine, 10% FBS, and 1% penicillin-streptomycin solution. THP1-Dual^TM^ (invivoGen, San Diego, CA, USA) cells are stably transfected with a secreted embryonic alkaline phosphatase gene (SEAP), which is under the control of an NF-κB-inducible promoter. They were maintained in Roswell Park Memorial Institute RPMI-1640 medium (Corning, Corning, NY, USA) with 10% FBS, 10 nM HEPES, and 1% penicillin/streptomycin solution. For handling procedures, a growth medium with Normocin 100 μg/mL and a medium with selective antibiotics, Blasticidin and Zeocin^®^ (invivoGen, San Diego, CA, USA), were used. Both HMC-3 and THP1-Dual^TM^ cells were grown under specific conditions in a humidified atmosphere incubator of 95% air and 5% CO_2_ at 37 °C. Cells were used at no more than seven passages.

### 4.2. Polygodial and Isotadeonal

Polygodial was obtained from the bark of *Drimys winteri* according to the protocols described by Paz et al., 2022 and Marín et al., 2022 [20,39]. Starting with 68 g of extract, an amount of 345 mg of purified polygodial was obtained. From this, an amount of 100 mg was used to obtain isotadeonal by epimerization in basic medium with sodium carbonate, producing 60 mg of isotadeonal after CC. Isotadeonal showed a retention factor (Rf) of 0.68 compared to polygodial, which had a Rf of 0.54 using hexane/EtOAc (4:1 *v*/*v*).

As polygodial and isotadeonal were not soluble in water, a stock solution was prepared in DMSO (dimethylsulfoxide) and further dilutions were carried out with culture media, resulting in the work solutions with DMSO at 0.1%. Briefly, 5.0 mg of each drimane terpene was dissolved in 50 μL of DMSO, obtaining a stock solution of 100 mg/mL for each compound, which were kept at −20 °C. From this solution, a dilution of 1000 times was performed in culture medium to obtain a concentration of 100 μg/mL of each compound, with a final concentration of DMSO at 0.1%. This last solution was filtered through a sterile filter of 0.22 μm.

### 4.3. Cell Viability Analysis in Response to Treatments

CellTiter 96^®^ Aqueous One Solution Cell Proliferation Assay (MTS) (Promega, Madison, WI, USA) was used to determine the toxic effect of polygodial and isotadeonal on HMC-3 cells. The MTS assay is based on the conversion of a tetrazolium salt into a colored aqueous soluble formazan product by mitochondrial activity from viable cells. The amount of formazan produced by dehydrogenase enzymes is directly proportional to the number of living cells in culture. The viability assays were performed according to the manufacturer’s protocols. Briefly, HMC-3 cells were placed into 96-well plates (5 × 10^3^ cells per well) in 100 μL and incubated at 37 °C. Then, cells were exposed to different concentrations of polygodial and isotadeonal (from 1.0 to 100 μM). After 24 h of incubation, 20 μL of MTS reagent was added to each well, followed by a 4 h incubation at 37 °C. The absorbance was measured by a microplate reader (VICTOR Nivo^TM^ Perkin Elmer, Waltham, MA, USA) at 490 nm. Results were expressed as the percentage of viability relative to the control.

### 4.4. Expression of NF-kB-SEAP

THP1-DualTM (100.000 cells/well) were seeded in 96-well plates and were pretreated with different concentrations of polygodial and isotadeonal (from 1 to 50 μM) for 30 min, followed by the addition of LPS (100 ng/mL) for 18 h. Alkaline phosphatase activity was determined in cell supernatants using a QUANTI-Blue mixture (InvivoGen, Toulouse, France), according to the manufacturer’s specifications. The absorbance was measured by a microplate reader (VICTOR Nivo^TM^ Perkin Elmer) at 655 nm and compared with positive control samples LPS (100 ng/mL). Subsequently, the percentage of inhibition was calculated, and the difference was compared with the sample stimulated only with LPS, which represented 100% of the activation of the pathway. Quercetin and CAPE (CAYMAN CHEMICAL COMPANY No. and No. 70750) were used as positive controls.

### 4.5. Western Blot Analysis

Protein expression was determined by Western blot analysis. HMC-3 cells (4 × 10^5^ cells/well) seeded in P60 plates were incubated in media for 12 h. Then, cells were exposed to 50 µM and 10 µM concentrations of polygodial and isotadeonal for 3 h and then stimulated with LPS (100 ng/mL) for 4 h. Cells were then rinsed twice with ice-cold PBS and lysed with RIPA Lysis Buffer System supplemented with 200 mM PMSF, Protease Inhibitor cocktail, and 100 mM Sodium Orthovanadate (ChemCruz, Huissen, The Netherlands). Lysates were cleared by centrifugation at 12,000 rpm for 20 min, and the supernatants were quantified using a Pierce^TM^ BCA Protein Assay Kit (Thermo Scientific^TM^, Waltham, MA, USA). Equal amounts of protein (30 µg) were heated at 95 °C for 5 min and separated using 10% SDS-PAGE. Following electrophoresis, the proteins were transferred onto a polyvinylidene fluoride membrane (PVDF, GVS Filter Technology, Morecambe, UK). The membranes were blocked with 5% non-fat dry milk at room temperature for 1 h and then incubated overnight at 4 °C with primary antibodies against IkB-α (44D4) Rabbit mAb #4812, p-IkB-α (Phospho-IkB-α (Ser32/36) (5A5) #9246 Mouse mAb, and α-Tubulin #2144 (Cell Signaling, Danvers, MA, USA). Immunoreactive bands were visualized using a horseradish peroxidase-conjugated secondary antibody (Anti-Mouse IgG, #715-035-150; Anti-Rabbit IgG 711-035-152, 1:10.000, Jackson Immunoresearch, West Grove, PA, USA). The bands were visualized by the SuperSignal^TM^ West Pico PLUS chemiluminescence substrate (Thermo Scientific^TM^, Waltham, MA, USA) in a photodocumenter (G: BOX Chemi XRQ gel doc system, SYNGENE, Bangalore, India) and band densitometry analysis was performed with Image J software V.1.49 (NIH, Bethesda, MD, USA). β-Actin was used as an internal control. Each sample was run and analyzed in triplicate.

### 4.6. In Silico Analysis

Molecular docking is essential for discovering potential drug candidates, elucidating molecular mechanisms, and supporting the development of novel therapeutic compounds. The crystal structure of IκB kinase β (IKKβ) complexed with the inhibitor K-252a (PDB ID: 4KIK) for docking analysis, was retrieved from the Protein Data Bank. The protein preparation process involved the removal of the bound inhibitor ligand and water molecules. Polar hydrogens were then added, and Kollman charges were applied. The 3D structures of isotadeonal, polygodial, and the reference standard quercetin were obtained from the PubChem database in SDF format. These ligands were then converted to PDB format using Open Babel software version 2.4.1 [40]. To optimize the ligand structures, UCSF Chimera software version 1.8 [41] was used for energy minimization. Subsequently, Gasteiger partial atomic charges (Q) were assigned, and the atom types were adjusted to Autodock4 (T)-compatible format using MGL Tools 1.5.6 before saving the structures in PDBQT format. Molecular docking was carried out using AutoDock Vina version 1.1.2 [42], focusing on hydrogen bonding interactions and binding affinities. The grid box resolution was configured at specific coordinates along the x, y, and z axes: 14.044, 33.258, and 75.955. Grid spacing was maintained at 0.375 Å, while grid dimensions were set at 56 × 50 × 50 Å, corresponding to the binding sites of K-252a in the target protein.

### 4.7. Pharmacokinetic Features—ADMET

The ADMET tool evaluates a drug’s characteristics prior to its development. Among these, toxicity is the most significant factor, leading to the failure of most drug candidates. Conducting ADMET/Tox studies is essential for ensuring the success of a drug candidate by identifying the most viable options [43]. The druggability and toxicological characteristics of the studied compounds, including their absorption, distribution, metabolism, excretion, and toxicity (ADMET), were analyzed using the publicly available SwissADME server (http://www.swissadme.ch/, accessed on 4 November 2024) and the ProTox-III platform (https://tox.charite.de/protox3/, accessed on 4 November 2024) [44,45]. The predicted parameters encompassed molecular weight (MW), the number of hydrogen bond acceptors (HBA) and donors (HBD), rotatable bonds, topological polar surface area (TPSA), partition coefficient (Consensus Log P), solubility class (ESOL), gastrointestinal (GI) absorption, status as a substrate or inhibitor of permeability glycoprotein (P-gp), permeability across the blood–brain barrier (BBB), skin permeability (Log Kp), and susceptibility to cytochrome P450 enzyme inhibition. These metrics were evaluated in alignment with Lipinski’s Rule of Five (RO5).

### 4.8. Statistical Analysis

All the experiments were repeated at least three times. The results were expressed as mean ± S.D., and the data were analyzed using one-way ANOVA followed by Dunnett’s test using Graphpad Prism Software Version 6.0 (GraphPad software, San Diego, CA, USA) to determine any significant differences. A *p* < 0.05 was considered statistically significant.

## 5. Conclusions

Polygodial and isotadeonal are active compounds found in the medicinal tree *Drimys winteri*. They were also reported to be present as the main bioactive compounds of *Polygonum punctatum,* a plant used in the traditional medicine of Brazil with anti-inflammatory activity, among others [46]. Polygodial has been shown to be an anti-inflammatory compound. At a dose of 12.8–128.1 μmol/kg, i.p., it inhibits paw edema in mice [47]. Moreover, it has been shown to inhibit the chronic inflammatory response to implants (polyurethane) in mice, reducing chemokines, cytokines, and producing an antifibrogenic effect [47]. In this research, we found that polygodial and its epimer isotadeonal inhibited the NF-κB signaling pathway by preventing the phosphorylation of IκBα in HMC-3 cells at 50 μM. Moreover, isotadeonal showed better activity than polygodial, reducing the secretion of alkaline phosphatase (SEAP) in an NF-κB reporter cell at 1 μM. The results of this study suggest that isotadeonal has potential in the control of neuroinflammatory processes, with higher potency than polygodial, CAPE, and quercetin. Moreover, its small and lipophilic structure provides advantages in crossing the BBB compared to polyphenols. However, further research is needed to elucidate its precise mechanisms of action in the neuroinflammatory process through in vivo experiments.

## Figures and Tables

**Table 1:** Docking binding energy and details of interactions of polygodial and isotadeonal docked in the active site of IKKβ (PDB: 4KIK).

Compound	Binding Energy (kcal/mol)	Number of Interactions	Interacting Amino Acids
Polygodial	−6.68	3	Cys99, Val152, Leu21
Isotadeonal	−7.12	4	Cys99, Val152, Leu21

**Table 2:** Binding interactions and positioning of polygodial and isotadeonal on IKKβ.

Compound	2D Binding Interactions	3D Pocket Positioning
Polygodial	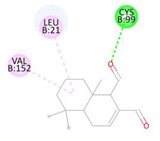	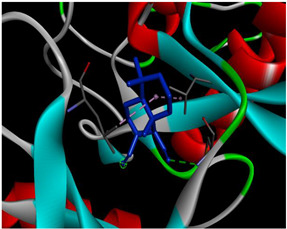
Isotadeonal	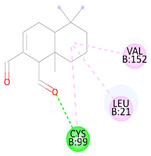	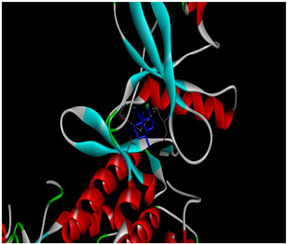

**Table 3:** Pharmacokinetics, druglikeness, and toxicity prediction of the major compounds.

Pharmacokinetics/Druglikeness	Isotadeonal	Polygodial
#Rotatable bonds	2	2
#H-bond acceptors	2	2
#H-bond donors	0	0
TPSA	34.14	34.14
Consensus Log P	2.93	2.89
ESOL Class	−3.20	−3.20
GI absorption	High	High
BBB permeant	Yes	Yes
Pgp substrate	No	No
CYP1A2 inhibitor	No	No
CYP2C19 inhibitor	No	No
CYP2C9 inhibitor	No	No
CYP2D6 inhibitor	No	No
CYP3A4 inhibitor	No	No
log Kp (cm/s)	−5.44	−5.44
Lipinski	Yes	Yes
Bioavailability score	0.55	0.55
Log VDss (human)	0.300	0.323
CNS permeability	−2.131	−2.131
Synthetic Accessibility	4.26	4.26
Toxicity		
AMES toxicity	No	No
Hepatotoxicity	No	No
hERG I/II inhibitors	No	No
Skin sensitization	Yes	Yes

## Data Availability

The original data presented in the study are openly available at https://doi.org/10.5281/zenodo.14845499.

## Supplementary Materials

The following supporting information can be downloaded at: https://www.mdpi.com/article/10.3390/molecules30071555/s1. NMR data and copies of spectra for polygodial and isotadeonal.

## Abbreviations

The following abbreviations are used in this manuscript:

ADMET	Absorption, Distribution, Metabolism, Excretion and Toxicity
BBB	Blood brain barrier
CAPE	Phenethyl ester of caffeic acid
CNS	Central nervous system
DMSO	Dimethylsulfoxide
ESOL	Solubility class
GI	Gastrointestinal
HBA	Hydrogen bond acceptors
HBD	Hydrogen bond donors
HMC-3	Human microglial cell clone 3
ICAM1	Intercellular adhesion molecule 1
IKK	IκB kinase
log P	Partition coefficient
LPS	Lipopolysaccharide
MIC	Minimum inhibitory concentration
MW	Molecular weight
NMR	Nuclear magnetic resonance
NF-κB	Nuclear factor-κB
P-gp	Inhibitor of permeability glycoprotein
RB	Number of rotatable bonds
Rf	Retention factor
SEAP	Secreted alkaline phosphatase
TLC	Thyn layer chromatography
TPSA	Predicted topological polar surface area
TRPA1	Transient receptor potential ankyrin 1
TRPV1	Transient receptor vanilloid 1
VCAM1	Vascular cell adhesion molecule 1
