# Supplementary material for: Inhibitory Potential of the Drimane Sesquiterpenoids Isotadeonal and Polygodial in the NF-kB Pathway
Source: Molecules. 2025 Mar 31;30(7):1555. doi: 10.3390/molecules30071555 (PMC11990674; doi:10.3390/molecules30071555)

Supporting Information

Contents:

NMR data and copies of spectra for polygodial S2

NMR data and copies of spectra for isotadeonal S8

Table S1. NMR Spectroscopic Data (^{1}H 600 MHz, ^{13}C 150 MHz, $CDCl_3$) for polygodial and comparison with reference data from the literature

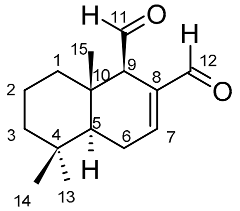

No	This work[a)]		Rodriguez et al.[b)]	
	δ_C (ppm)	δ_H (ppm, m (*J* (Hz))	δ_C (ppm)	δ_H (ppm); m (*J* (Hz))
1	39.7	1.84 (dm, 13.0)<br>1.38 (ddd, 13.0, 13.0, 4.1)	39.5	1.36 (td)<br>1.82 (dddd)
2	18.1	1.49 (m)<br>1.49 (m)	18.0	1.47 (m)<br>1.52 (qt)
3	41.8	1.49 (m)<br>1.23 (ddd, 12.3, 12.3, 4.3)	41.7	1.21 (m)<br>1.42 (m)
4	33.2	--	33.1	--
5	49.1	1.26 (dd, 12.1, 4.5)	48.9	1.24 (dd)
6	25.3	2.50 (dm, 20.4)<br>2.31 (dddd, 20.4, 12.1, 3.9, 2.4)	25.2	2.49 (dddd)<br>2.30 (dddd)
7	154.4	7.13 (dt, 5.6, 2.3)	154.3	7.12 (ddd)
8	138.4	--	138.2	--
9	60.4	2.82 (m)	60.2	2.80 (dddd)
10	37.0	--	36.8	--
11	202.1	9.53 (d, 4.5)	202.0	9.51 (d)
12	193.4	9.45 (s)	192.8	9.44 (s)
13	33.2	0.92 (s)	33.1	0.90 (s)
14	22.1	0.95 (s)	21.9	0.94 (s)
15	15.4	0.94 (s)	15.2	0.93 (s)

[a)] ^{1}H NMR (600 MHz, $CDCl_3$); ^{13}C NMR (150 MHz, $CDCl_3$). b) ^{1}H NMR (400 MHz, $CDCl_3$); ^{13}C NMR (100 MHz, $CDCl_3$); Rodríguez, B.; Zapata, N.; Medina, P.; Viñuela, E. A complete 1H and 13C NMR data assignment for four drimane sesquiterpenoids isolated from *Drimys winterii*. *Magn. Reson. Chem.* **2005**, *43*, 82-84, doi 10.1002/mrc.1500.

Figure S1: ^{1}H NMR (500 MHz, $CDCl_3$) of polygodial

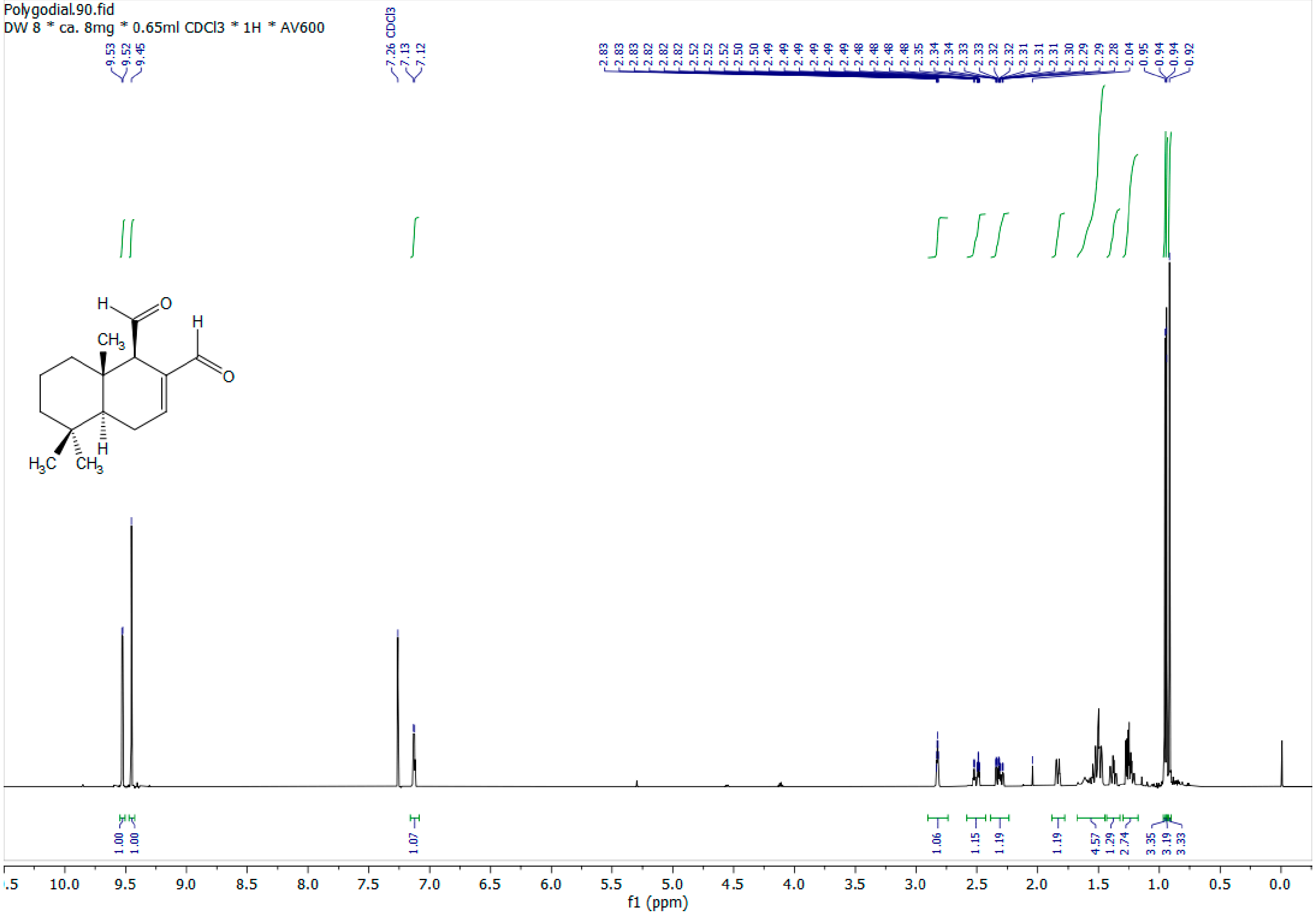

Figure S2: $^{13}C\{^1H\}$ NMR (125 MHz, $CDCl_3$) of polygodial

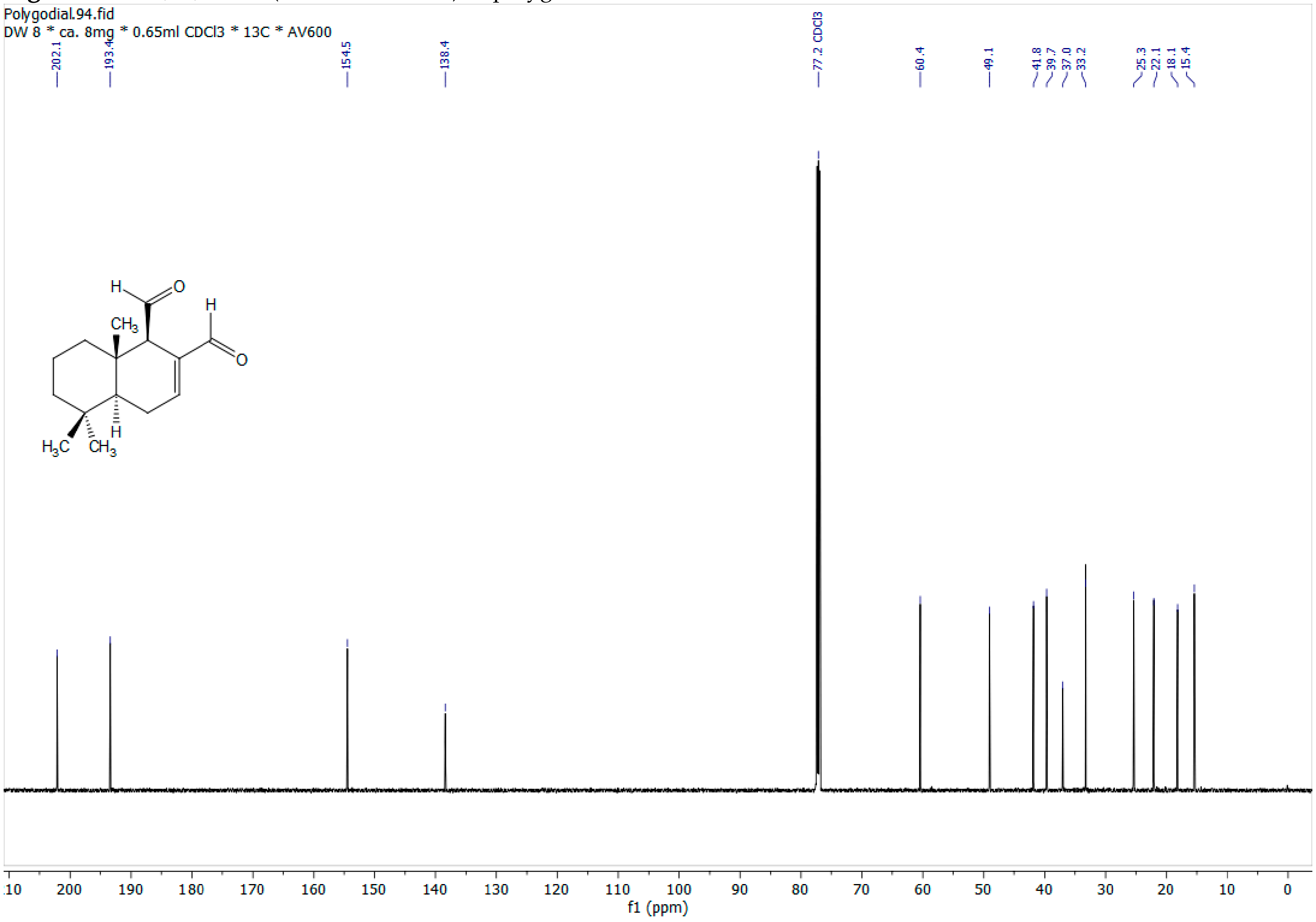

Figure S3: H,H-COSY (500 MHz, $CDCl_3$) of polygodial

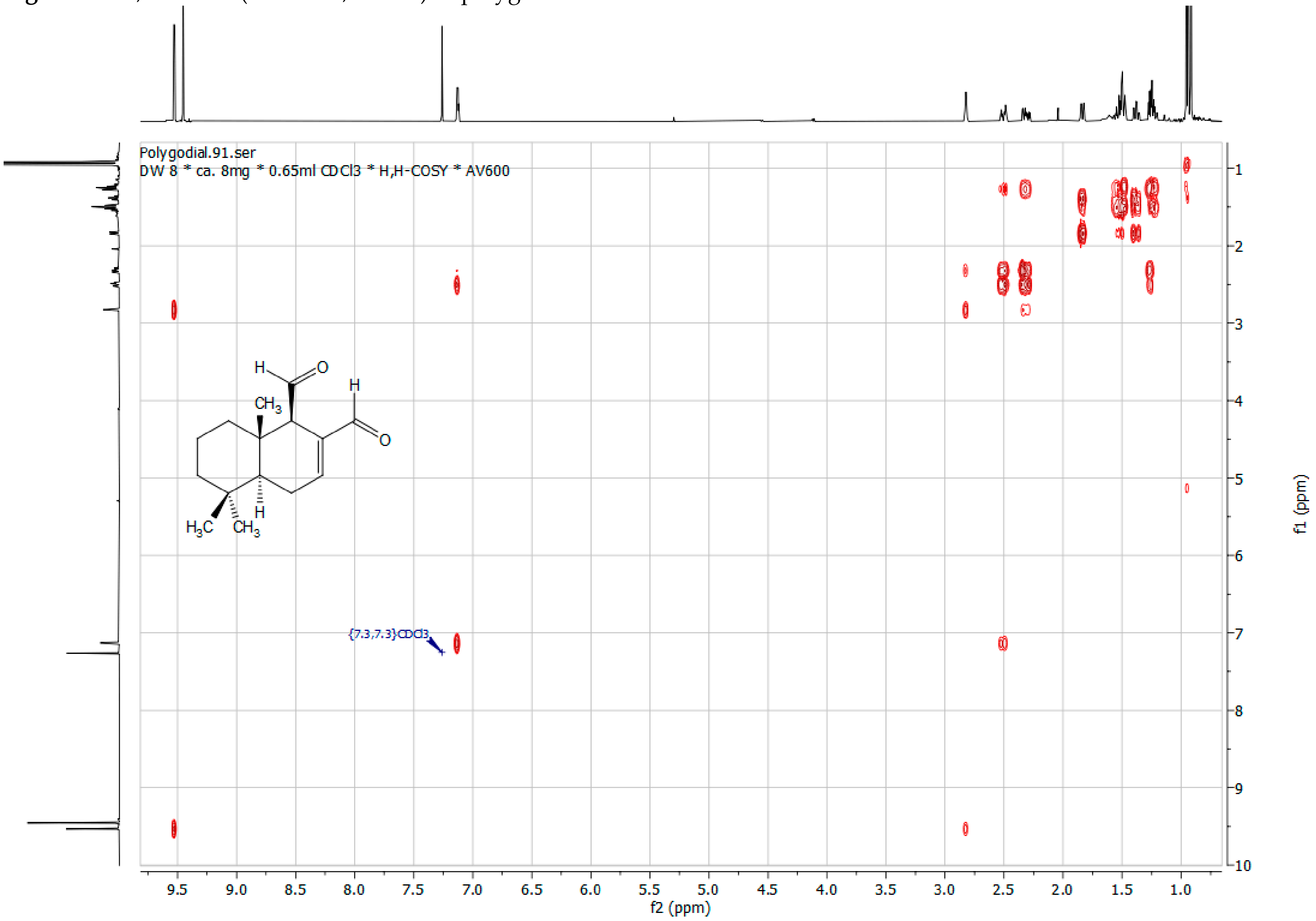

Figure S4: HSQC (500/125 MHz, CDCl3) of polygodial

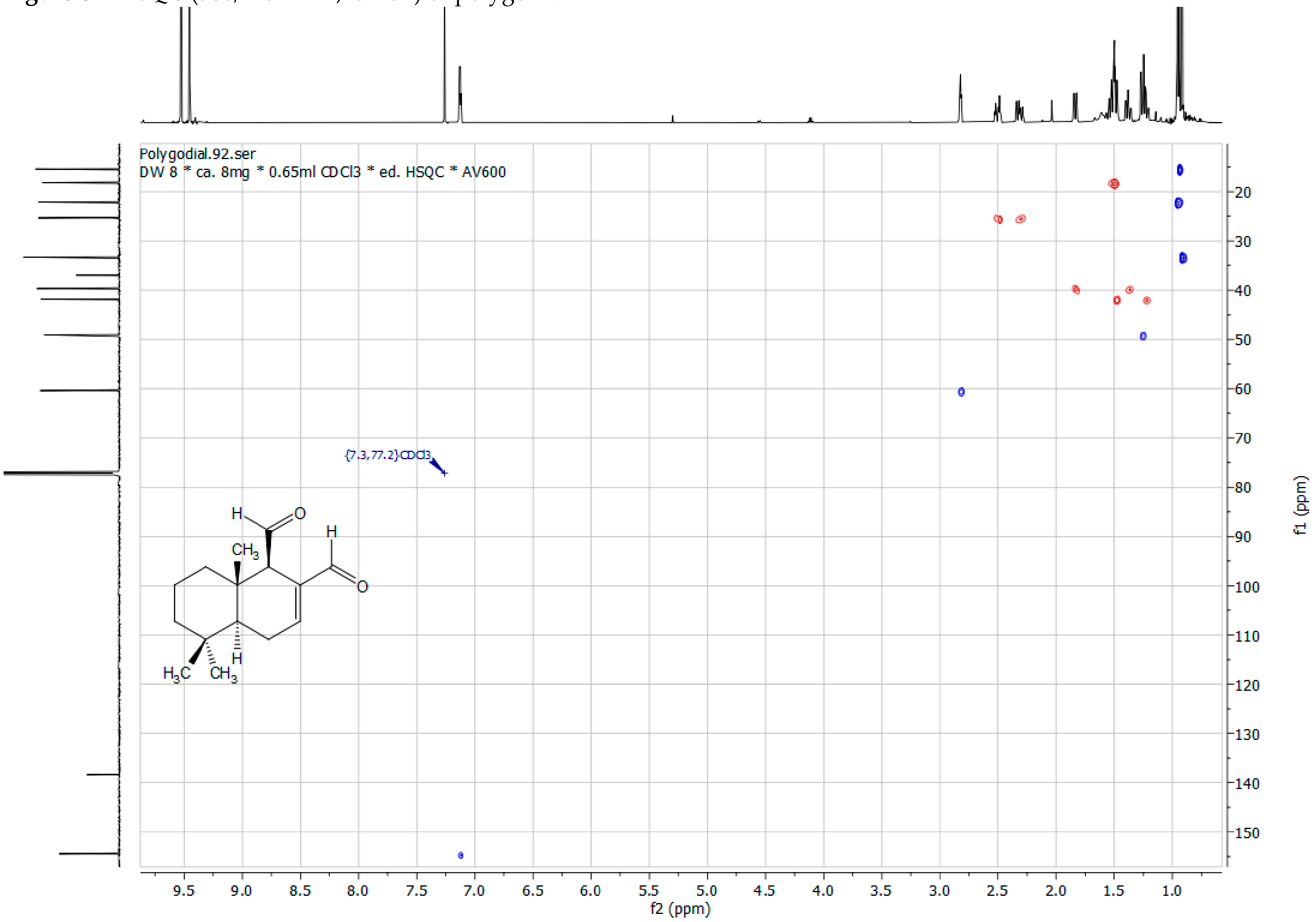

Figure S5: HMBC (500/125 MHz, $CDCl_3$) of polygodial

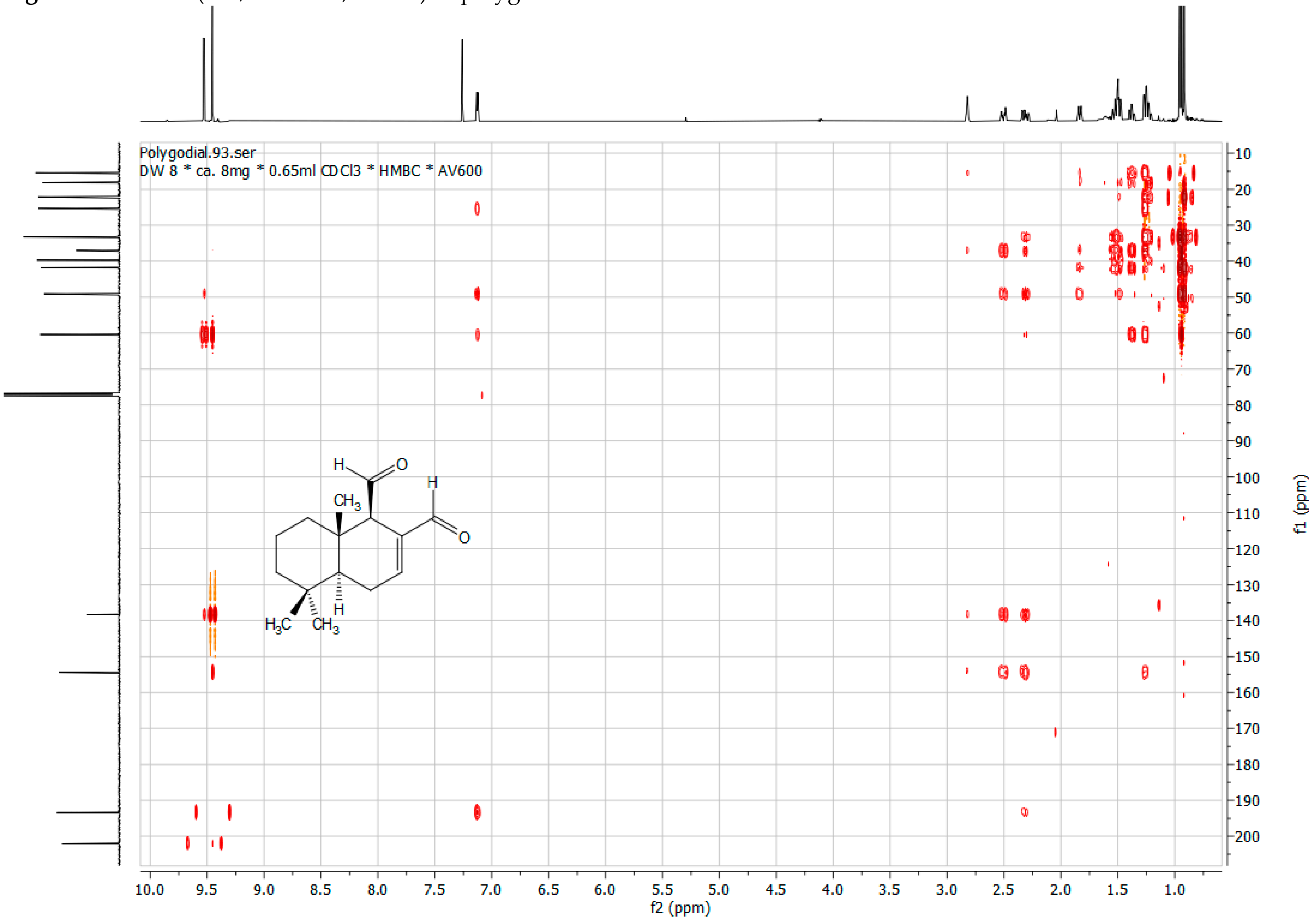

Table S2. NMR Spectroscopic Data (^{1}H 500 MHz, ^{13}C 150 MHz, $CDCl_3$) for isotadeonal and comparison with reference data from the literature

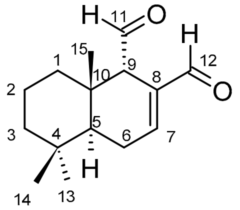

No	This work[a)]		Rodriguez et al.[b)]	
	δ_C (ppm)	δ_H (ppm, m (*J* (Hz))	δ_C (ppm)	δ_H (ppm); m (*J* (Hz))
1	37.3	1.45 (m)<br>1.77 (dm, 12.6)	37.1	1.44 (m)<br>1.76 (dddd)
2	18.5	1.49 (m)<br>1.62 (dt, 13.5, 2.9)	18.3	1.45 (m)<br>1.60 (m)
3	42.2	1.15 (ddd, 13.7, 13.7, 3.9)<br>1.47 (m)	42.0	1.14 (ddd)<br>1.42 (m)
4			32.8	--
5	44.4	1.55 (dd, 11.7, 5.1)	44.1	1.54 (ddq)
6	25.7	2.55 (dt, 20.5, 5.1)<br>2.21 (ddm, 20.5, 11.7)	25.5	2.54 (dt)<br>2.19 (dddd)
7	153.6	7.09 (dd, 5.0, 2.7)	153.5	7.08 (ddt)
8	137.5	--	137.3	--
9	58.6	3.25 (dd, 2.6, 2.6)	58.4	3.23 (dddd)
10	37.8	--	37.6	--
11	202.3	9.84 (d, 2.6)	202.3	9.83 (dd)
12	192.9	9.40 (s)	192.8	9.38 (d)
13	32.8	0.90 (s)	32.7	0.89 (s)
14	22.0	0.92 (s)	21.8	0.91 (s)
15	21.6	0.95 (m)	21.4	0.94 (dd)

[a)] ^{1}H NMR (500 MHz, $CDCl_3$); ^{13}C NMR (150 MHz, $CDCl_3$). b) ^{1}H NMR (400 MHz, $CDCl_3$); ^{13}C NMR (100 MHz, $CDCl_3$); Rodríguez, B.; Zapata, N.; Medina, P.; Viñuela, E. A complete 1H and 13C NMR data assignment for four drimane sesquiterpenoids isolated from *Drimys winterii*. *Magn. Reson. Chem.* **2005**, *43*, 82-84, doi 10.1002/mrc.1500.

Figure S6: ^{1}H NMR (500 MHz, $CDCl_3$) of isotadeonal

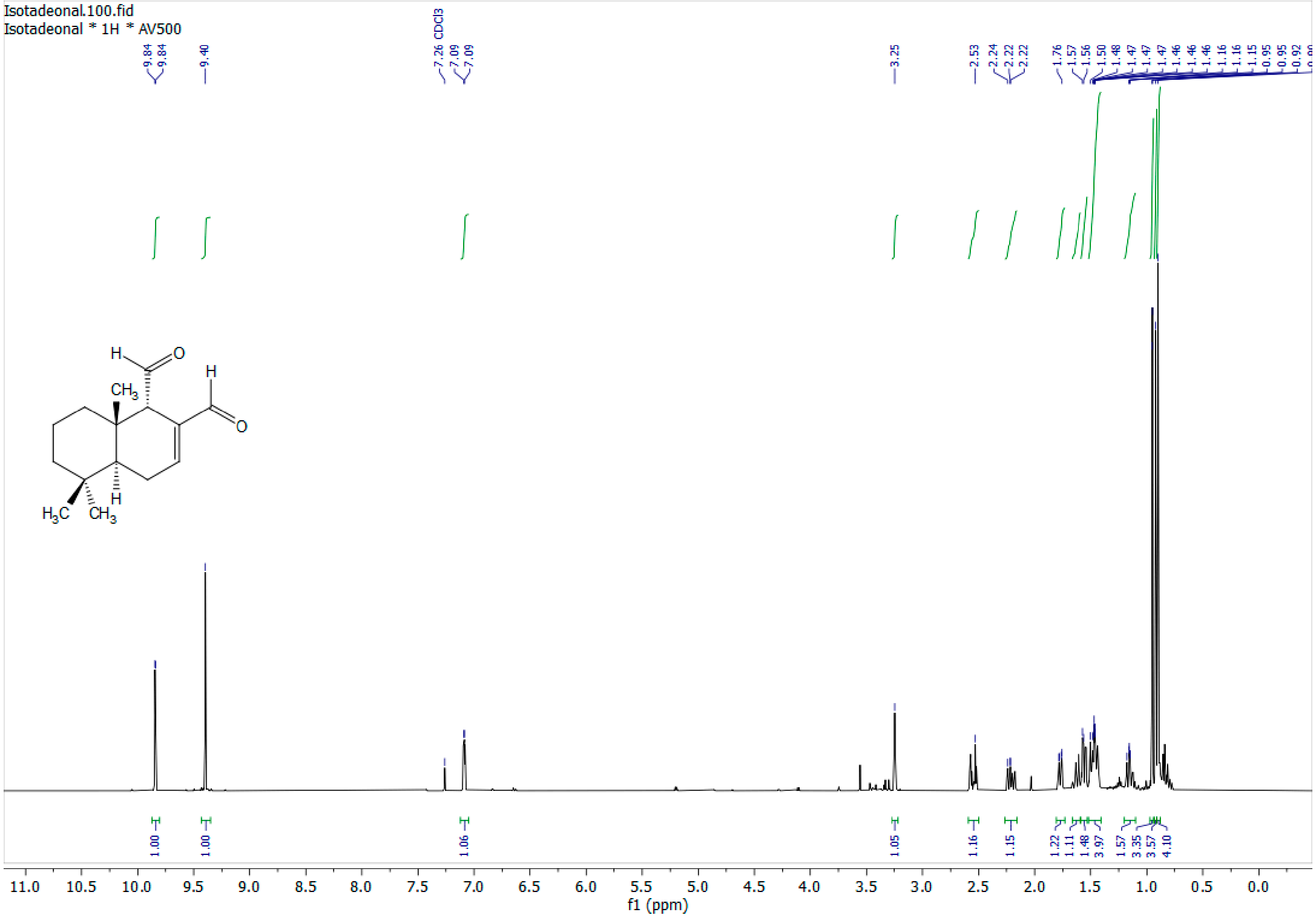

Figure S7: $^{13}C\{^{1}H\}$ NMR (150 MHz, $CDCl_3$) of isotadeonal

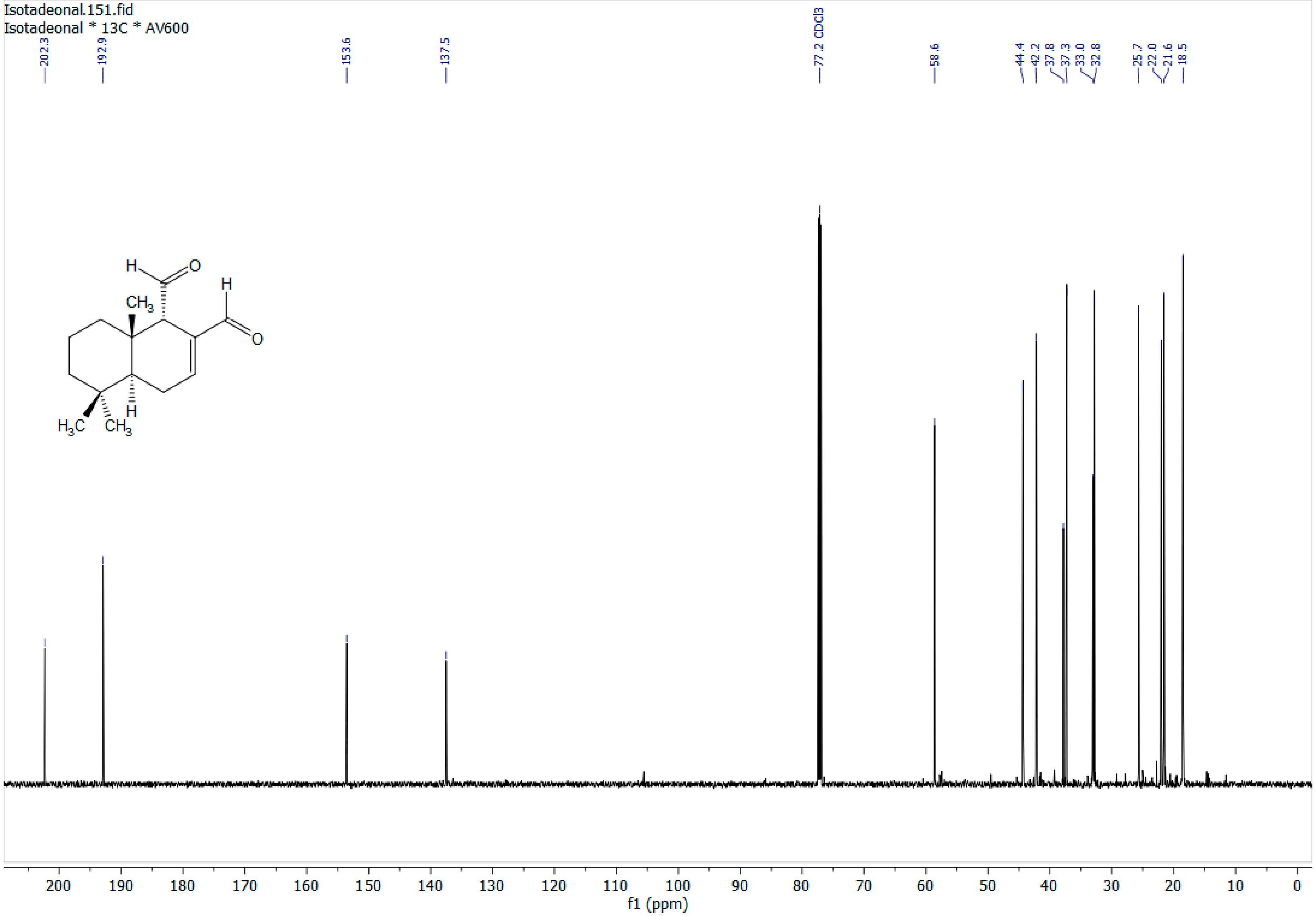

Figure S8: H,H-COSY (500 MHz, $CDCl_3$) of isotadeonal

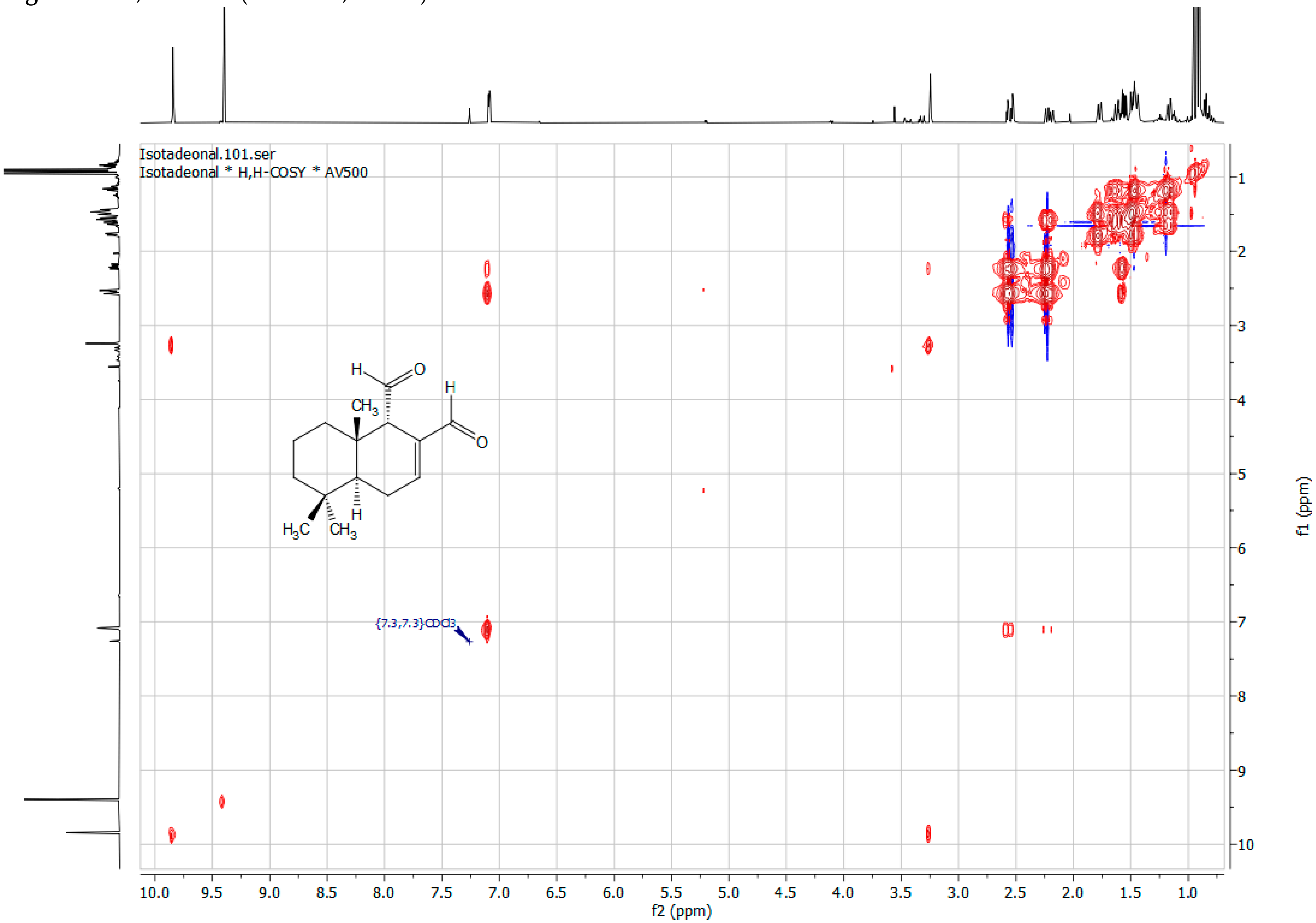

Figure S9: HSQC (500/125 MHz, $CDCl_3$) of isotadeonal

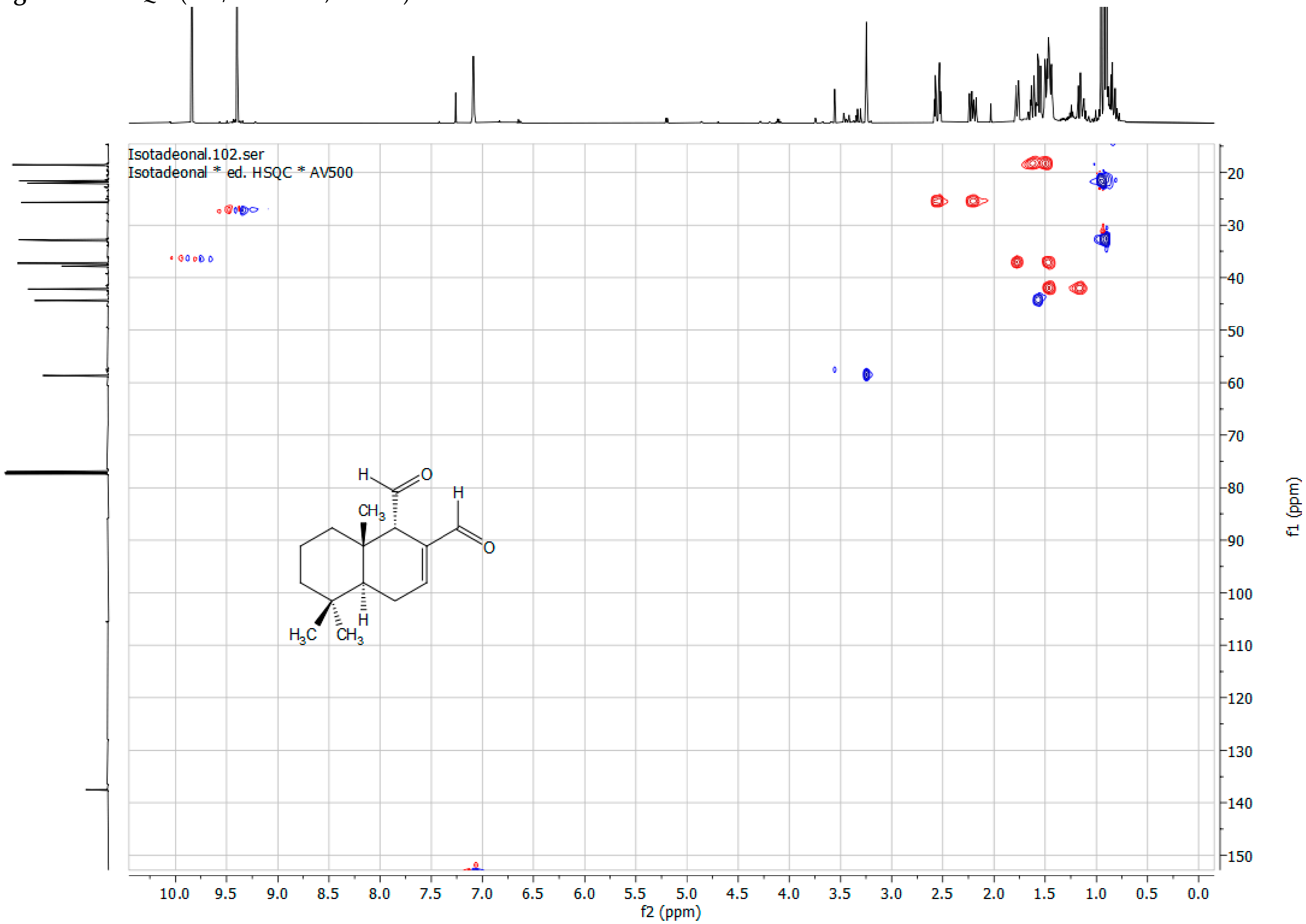

Figure S10: HMBC (500/125 MHz, $CDCl_3$) of isotadeonal

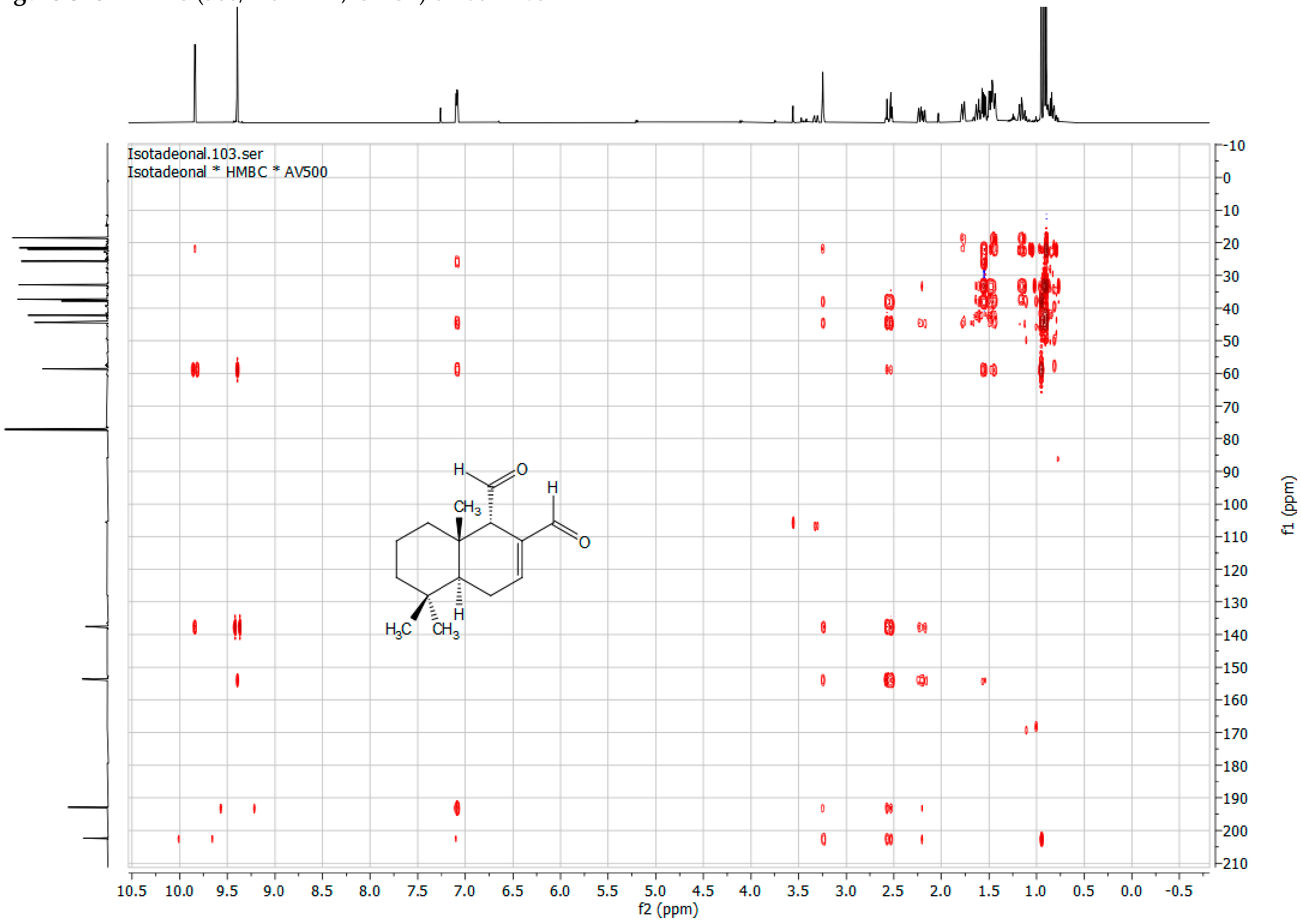

Figure S11: NOESY (500 MHz, $CDCl_3$) of isotadeonal

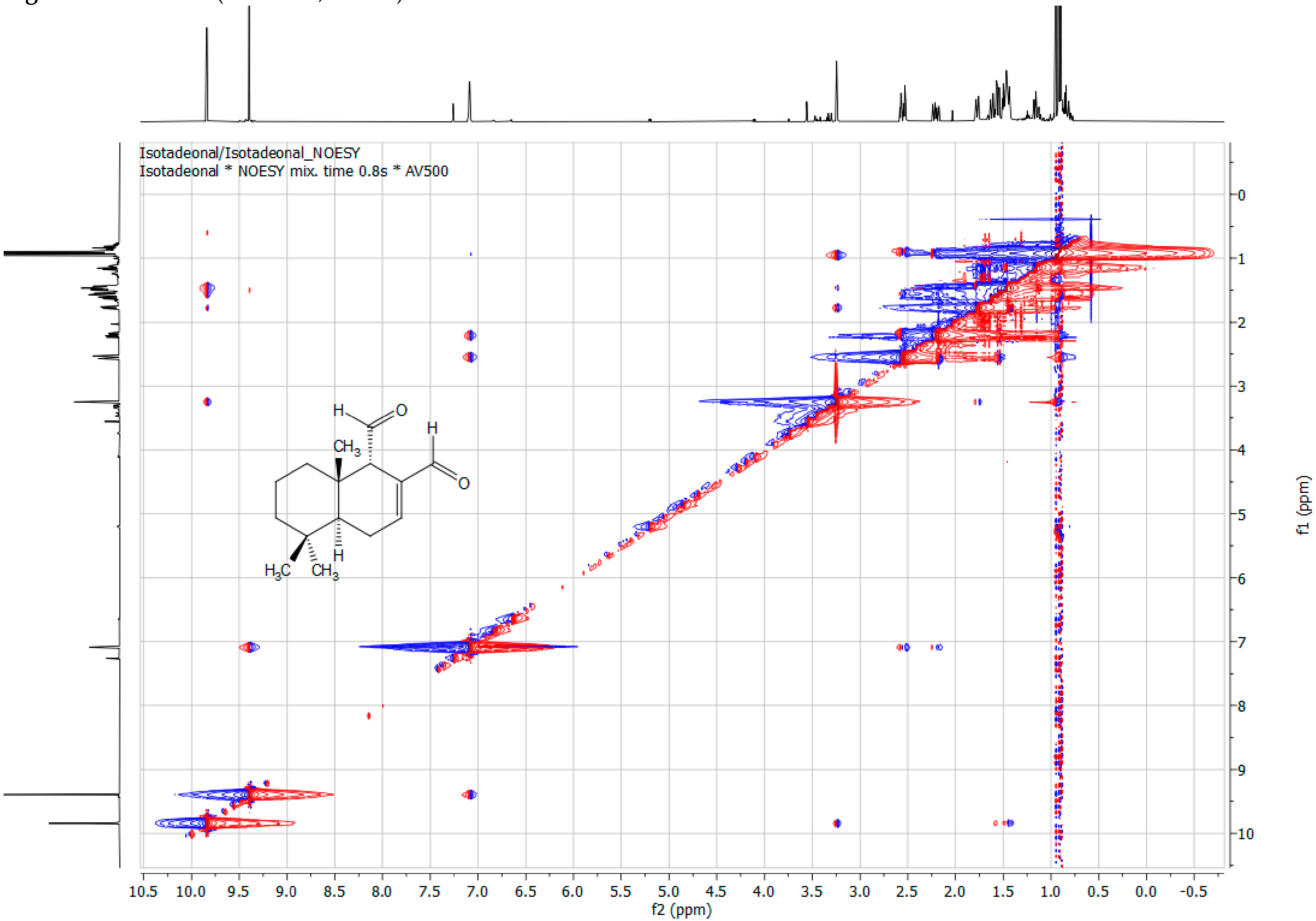

Supplement: Supplementary file 1 [file molecules-30-01555-s001.zip › molecules-3501076-supplementary.pdf]
